# Performance Implications of Divergent Shell Size Preferences and Exoskeleton Mass of Two Closely Related Hermit Crabs

**DOI:** 10.1002/ece3.73044

**Published:** 2026-02-04

**Authors:** Chloe B. MacLean, Louis A. Gosselin

**Affiliations:** ^1^ Department of Biological Sciences Thompson Rivers University Kamloops Canada

**Keywords:** adaptations, desiccation, heat stress, intertidal stressors, microhabitat use, motility, tolerance thresholds, trait divergence

## Abstract

Hermit crabs use gastropod shells for protection from abiotic stressors and predators. However, two sympatric species of hermit crab, *Pagurus hirsutiusculus* and *Pagurus granosimanus* , have divergent shell size preferences. Differences in shell size use were evident in the field: for a given body mass, *P. granosimanus* used shells that were 136%–300% larger than *P. hirsutiusculus* . The present study examined the possible morphological adaptations associated with the shell size preferences of *P. hirsutiusculus* and *P. granosimanus* as well as the costs and benefits associated with the preference of *P. hirsutiusculus* for shells that are too small to enclose and protect the entire body of the crab. When exposed to desiccation conditions commonly encountered during low tide emersion, *P. hirsutiusculus* using large shells survived much longer than individuals using small shells. And in motility trials, *P. hirsutiusculus* moved significantly faster when using a small shell than when using a large shell. It was therefore hypothesized that *P. hirsutiusculus* might produce a heavier exoskeleton than *P. granosimanus* to compensate for the reduced protection obtained by *P. hirsutiusculus* from small shells. Our findings support this hypothesis: relative to body mass, the carapace was 15%–90% heavier in *P. hirsutiusculus* than in *P. granosimanus* , a difference further confirmed by the claw mass, which was 59%–81% heavier in *P. hirsutiusculus* than in *P. granosimanus* . The use of larger shells in *P. granosimanus* provides enhanced protection, allowing for the production of a lighter exoskeleton, but likely imposes reduced motility and increased energetic cost. *Pagurus hirsutiusculus* , on the other hand, uses smaller, lighter shells that impose lesser energetic costs and allow greater motility but leave the animal more vulnerable to stressors; *P. hirsutiusculus* compensates for this increased vulnerability by producing a heavier carapace and claws. The differences in shell size and exoskeleton mass suggest ecological implications for these species, particularly with regard to microhabitat use.

## Graphical abstract

Two co‐occurring species of hermit crabs use very different shell sizes, one species using shells that are too small to accommodate all the carapace or claws. The two crab species were found to have diverged in their adaptations to their environment: one species adopting a suite of traits, including the use of large shells, that enhance protection from environmental stressors at the cost of reduced motility and increased energetic expenses, the other species adopting traits maximizing motility and low energetic expenses at the cost of reduced structural protection.

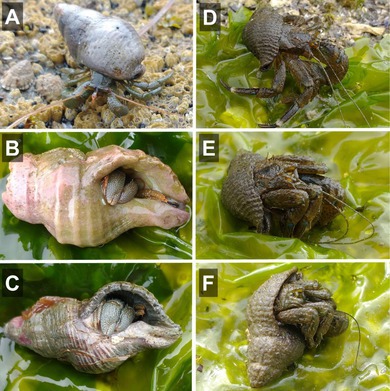

## 1 Introduction

Hermit crab performance is largely dependent on the features of the gastropod shells they use. In particular, the type (i.e., snail species that produced the shell) and size of shells used by hermit crabs have been found to influence hermit crab survivorship (Angel 2000), growth (Bertness 1981a; Angel 2000), fecundity (Bertness 1981a), and motility (Bach and Hazlett 2009). Accordingly, hermit crabs are selective for shell size (Angel 2000; Mantelatto et al. 2007; Straughan and Gosselin 2014), shell mass (Briffa and Elwood 2005), and shell type (Vance 1972; Straughan and Gosselin 2014). Also, as hermit crabs grow in size, they must repeatedly find larger and, in some cases, different types of shells to occupy (Turra and Leite 2004; Straughan and Gosselin 2014). Thus, the shell represents a resource that strongly affects hermit crab survival and growth throughout ontogeny as well as reproduction.

Hermit crabs use shells for protection from predation and abiotic stressors (Hazlett 1981). For instance, thick shells can provide the hermit crab protection from predation and water loss (Taylor 1981; Bertness 1981b; Alcaraz et al. 2015). However, the use of gastropod shells also involves costs, such as the energy expended carrying the shell, leaving the crab with less energy to invest in survival, growth, and reproduction (Herreid and Full 1986). Hermit crabs must therefore balance the costs and benefits of shell use. For example, the intertidal hermit crab *Pagurus granosimanus* has been shown to change shells less frequently when exposed to chemical cues from a predator and from dead conspecifics (Bulinski 2007); shells are changed less frequently to avoid the cost of increased vulnerability to predation that occurs due to the soft abdomen being exposed during shell exchange. Moreover, in the presence of the swimming crab *Arenaeus mexicanus*, a natural predator, the intertidal hermit crab *Calcinus californiensis* has been found to select larger shells (Arce and Alcaraz 2013); although larger shells are more energetically costly to carry, the benefits of increased protection against the predator would outweigh the increased energetic costs. Therefore, hermit crabs must balance the costs and benefits of shell use in response to the ambient conditions in their environment.

**FIGURE 1:**
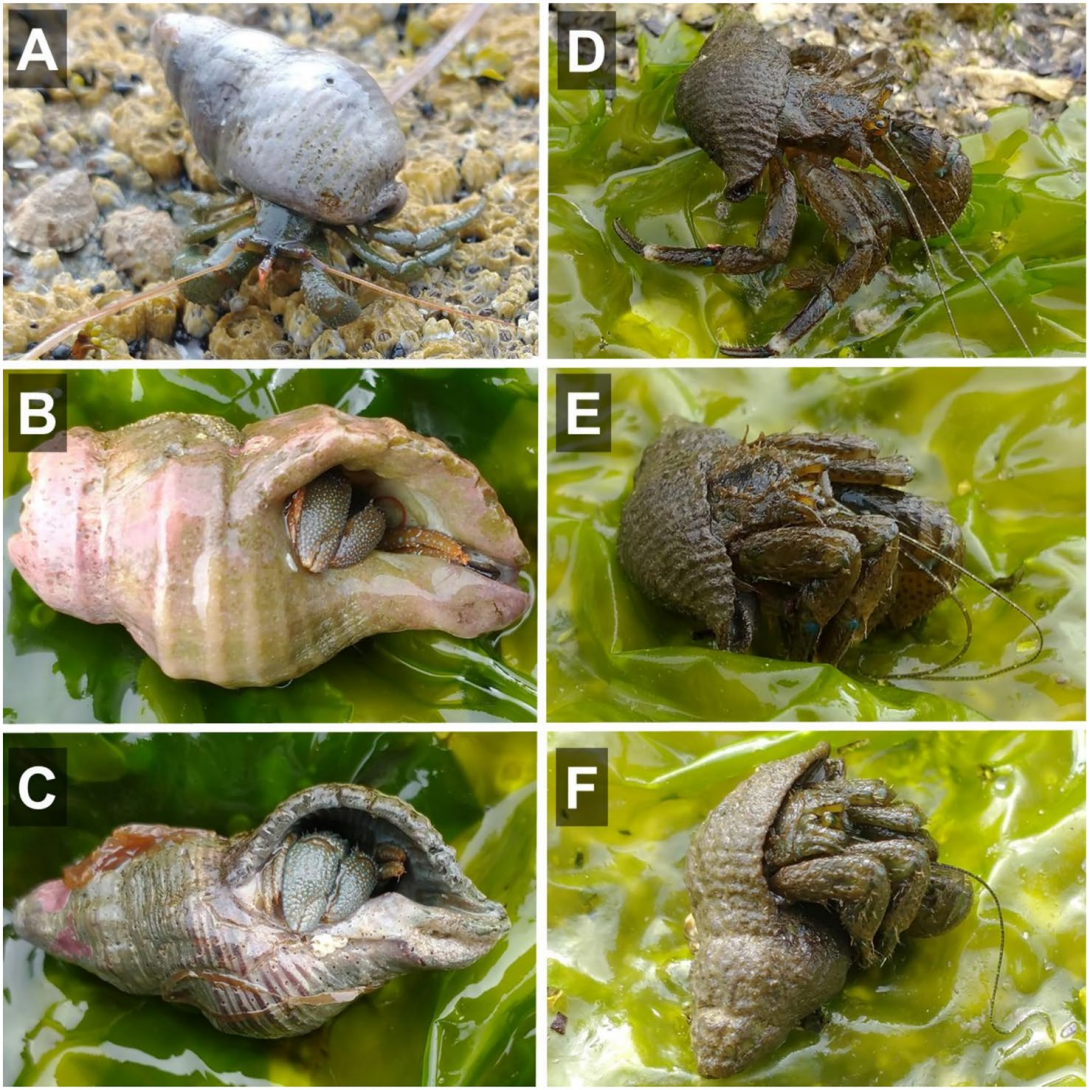
Comparative body exposure of *Pagurus granosimanus* (A–C) and *Pagurus hirsutiusculus* (D–F) in the field. Individuals are shown while extended and active (A, D) and fully retracted (B–C, E, F), illustrating the substantially greater degree of exposure of the carapace and appendages in *P. hirsutiusculus* when retracted.

Two closely related species of hermit crab, *P. granosimanus* and *Pagurus hirsutiusculus* , share the same intertidal habitats (Bollay 1964; Vance 1972; Spight 1977) from Alaska to California (Jensen 2005). *Pagurus granosimanus* and *P. hirsutiusculus* grow through similar size ranges but select different shell types throughout ontogeny, minimizing interspecific competition (Straughan and Gosselin 2014). Both species occur in tidepools and on rocky substrata, and although their intertidal ranges overlap greatly, *P. hirsutiusculus* can be found higher in the intertidal zone than *P. granosimanus* (Abrams 1987a, 1987b). Resource and habitat partitioning between these two species results in much stronger intraspecific competition than interspecific competition (Vance 1972; Abrams 1987a, 1987b). Additionally, *P. hirsutiusculus* is reported to prefer small shells that do not fully protect their entire body, while *P. granosimanus* select shells that are large enough to provide full body protection (Vance 1972; Straughan and Gosselin 2014). The shell size preference of *P. hirsutiusculus*, often leaving the head region of the body exposed (Figure 1), is intriguing because it seems inconsistent with the idea that body protection is the primary purpose of shell use and that hermit crabs seek shells with a high internal volume‐to‐shell mass ratio (Alcaraz et al. 2015). The divergent shell size preferences of *P. hirsutiusculus* and *P. granosimanus* (Figure 1) suggest they have evolved different strategies to balance the costs and benefits of shell use; however, the costs and benefits associated with the use of small shells have not yet been determined in *P. hirsutiusculus*, nor is it known how *P. hirsutiusculus* and *P. granosimanus* have morphologically adapted to compensate for their divergent shell preferences. Given that shells can protect hermit crabs from environmental stress, understanding the functional consequences of the use of small shells by *P. hirsutiusculus* and the morphological adaptive mechanisms that allowed *P. hirsutiusculus* and *P. granosimanus* to diverge in their shell size preferences can provide insight into how hermit crab populations may respond to future changes in their environment.

The purpose of this study was to explore the functional costs of the preference for small shells in *P. hirsutiusculus* and the morphological adaptive strategies associated with the divergent shell preferences of *P. hirsutiusculus* and *P. granosimanus*. This was accomplished by addressing four specific objectives: (1) to verify the previous claim that *P. hirsutiusculus* and *P. granosimanus* in the field use different shell sizes relative to their body mass (Straughan and Gosselin 2014); and also to test the following hypotheses: (2) that smaller shells confer less protection from desiccation to *P. hirsutiusculus* than larger shells; (3) that smaller shells enable greater motility of *P. hirsutiusculus* than larger shells; and (4) that *P. hirsutiusculus* produces a heavier carapace than *P. granosimanus* to compensate for the reduced protection obtained by *P. hirsutiusculus* from small shells.

## 2 Methods

**FIGURE 2:**
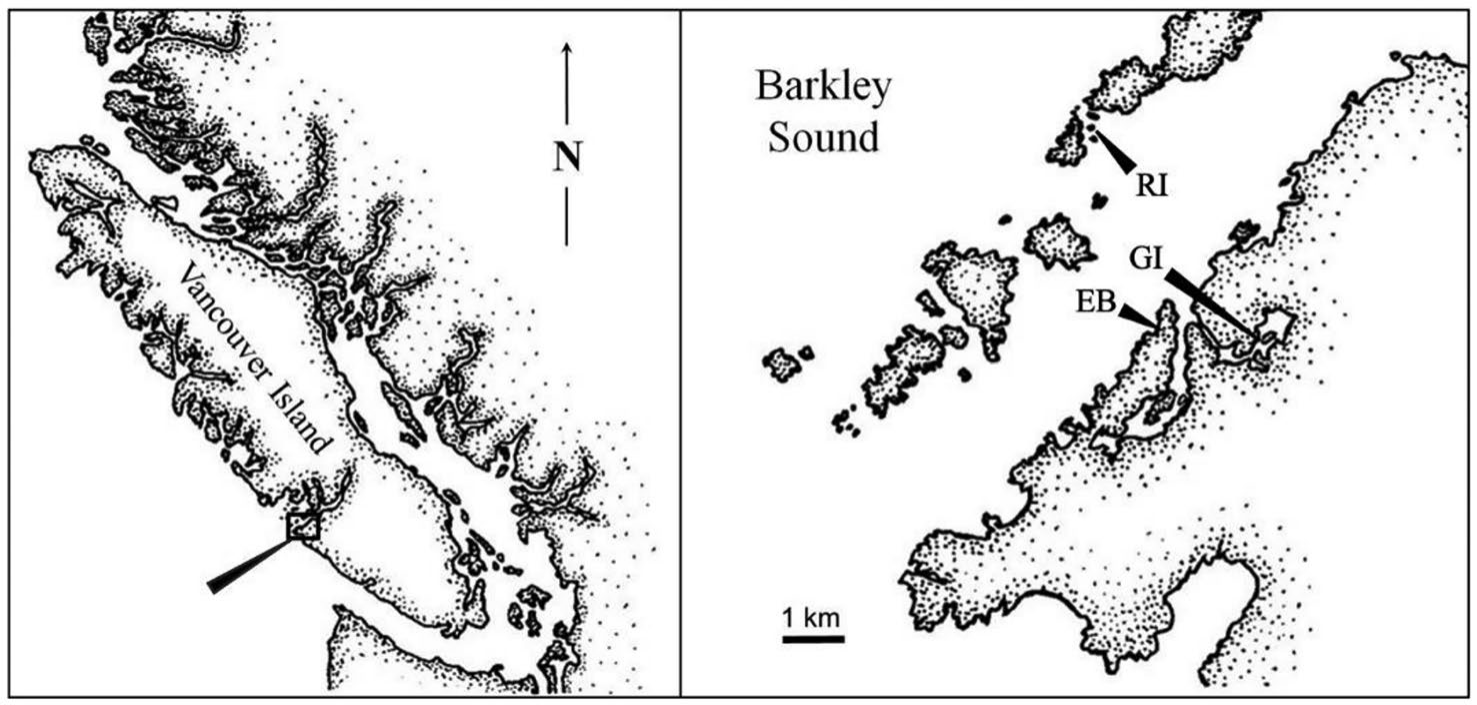
Field sites within Barkley Sound: Grappler Inlet (GI), Ross Islets (RI), and Eagle Bay (EB). Map modified from Gosselin and Chia (1995).

This study was carried out from May to August 2022 at the Bamfield Marine Science Centre (BMSC) and at three field sites in Barkley Sound, on the west coast of Vancouver Island: Grappler Inlet (48°49′54″ N, 125°07′05″ W), Ross Islets (48°52′19″ N, 125°09′42″ W), and Eagle Bay (48°50′12″ N, 125°08′36″ W) (Figure 2). Abundant populations of *Pagurus hirsutiusculus* and *Pagurus granosimanus* inhabit the mid‐ to upper intertidal zone at all three sites. All three field sites contain similar microhabitats, including tidepools, rock crevices, and rocky substrates, while sandy substrates are also present at Grappler Inlet and Ross Islets.

### 2.1 Shell Size Use in the Field

*Pagurus hirsutiusculus* and *P. granosimanus* from our three field sites were examined to determine the relationship between hermit crab body mass and the size of the shell they use in the field. At each field site, 25 individuals of both species were sampled randomly within a 10–20 m stretch of intertidal shoreline from microhabitats where both species commonly occur. Sampling across overlapping body mass ranges ensured consistent sampling effort across field sites. Morphometric data, including hermit crab body mass and shell length, were recorded from 25 individuals of each species at each site. This data was used to determine if the interspecific divergence in shell sizes used in the field previously reported by other studies was also occurring at our field sites. The cube of the shell length (SL^3^) was used as an index of shell volume, which allows relative shell volume comparisons between hermit crab species. In addition, given that shell size preferences can change as a hermit crab grows in body mass (BM) (Straughan and Gosselin 2014), this was accounted for by calculating the ratio of SL^3^ × BM^−1^ for the purpose of comparisons between species. For each field site, a two‐sample *t*‐test was performed to determine if the SL^3^ × BM^−1^ ratio differed between the two species. Claw lengths of these hermit crabs are reported in Table S1.

### 2.2 Vulnerability to Desiccation

#### 2.2.1 Determination of Temperature Tolerance Thresholds

To determine the temperature to be used in the desiccation tolerance experiment, a preliminary experiment was carried out to determine the range of temperatures and exposure durations that are nonlethal to air‐exposed *P. hirsutiusculus* . To do this, temperature trials were carried out in a temperature‐controlled incubator at each of the following temperatures: 20°C, 22°C, and 25°C. These three air temperatures were selected for the temperature tolerance trials because they occur frequently in the intertidal zone throughout the summer in Barkley Sound (Jenewein and Gosselin 2013).

Twenty *P. hirsutiusculus* with a claw length of 6–7 mm, the same size range used in all subsequent experiments in this study, were collected from Grappler Inlet, and three additional body parameters were also recorded for each individual: shell type (snail species), shell length, and crab body mass including the shell. These hermit crabs, in their original shells, were then submerged in seawater for about 3 s to coat the animal and its shell with seawater. Each crab was then individually placed in a small container (15.5 × 15.5 × 5 cm), and each container also received a cloth pad soaked in seawater to maintain a high relative humidity, as close to 100% as possible, throughout the experiment. Eight of the 20 containers also received a temperature datalogger (iButton model DS1921) to monitor temperature at 10 min intervals. The containers were then sealed, and all containers were placed together in a large, uncovered bin (60 × 40.5 × 34.5 cm); the bin was then placed in an incubator set at the desired temperature.

Within the incubator, air circulation and a homogenous temperature were maintained using two small fans, positioned to circulate air into the bin, around all the containers. Every 2 h, the bin was briefly removed from the incubator for ~2 min, and each container was opened to determine whether the crab was inert or active; opening the containers also served to replenish the oxygen in each container. If the crab was noticeably moving, it was recorded as active and the container was closed. If the crab was not moving, it was gently probed; if the crab did not react to probing, it was recorded as inert. Once a first crab was found to be inert, a humidity datalogger (iButton model DS1923) was placed in its container to monitor relative humidity; humidity dataloggers monitor humidity through a small opening that must not be disturbed or obstructed, so a humidity datalogger could not be placed in a container with an active hermit crab, as the crab might interfere with the datalogger. Once 50% of the crabs had become inert, the temperature experiment was ended and each of the 20 crabs was transferred to a separate mesh cage and submerged in seawater at 11°C. After 12 h of submersion, each crab was inspected again to determine if it was dead or alive.

#### 2.2.2 VPD Levels in the Field

Intertidal vapor pressure deficit (VPD) levels (Jenewein and Gosselin 2013) were quantified at the Grappler Inlet field site to characterize the desiccation levels experienced by hermit crabs in their natural habitat and also to inform the design of the desiccation tolerance experiment. On the morning of 31 July 2022, a warm sunny day, three humidity dataloggers were placed in the intertidal zone among microhabitats where both species of hermit crab can be found. One datalogger was placed on a rock in a fully exposed location, a second datalogger was tucked under a large rock that provided shade and minimal airflow, and a third datalogger was placed inside a highly sheltered rock crevasse. The dataloggers recorded relative humidity at 5 min intervals for 3 h 50 min; the dataloggers were then recovered just before being submerged by the incoming tide. Since it takes a few minutes for humidity dataloggers to adjust to ambient relative humidity levels, the first 15 min of data were not used.

#### 2.2.3 Effect of Shell Size on Vulnerability to Desiccation

**FIGURE 3:**
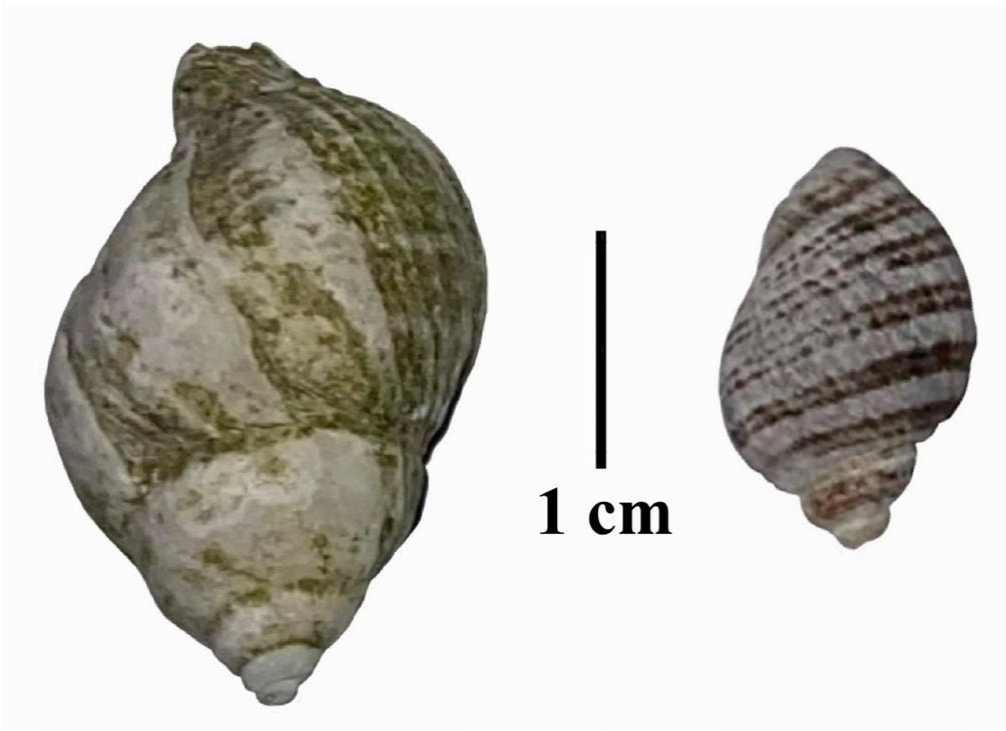
Large shell ( *N. lamellosa* ) and small shell (*N. ostrina*) used in the desiccation and motility experiments (scale bar = 1 cm).

The response of *P. hirsutiusculus* to desiccation was examined to understand the effect of using a small shell on vulnerability to desiccation. Prior to starting a desiccation trial, 10 *P. hirsutiusculus* with a claw length of 6–7 mm were collected from Grappler Inlet. Each crab was deshelled by holding the shell between the thumb and index finger; the heat from the hand as well as the inability to crawl away with its shell enticed the crab to abandon its shell. If this deshelling method did not work, the apex of the shell was held on a warm water‐soaked cloth in a fingerbowl until the crab exited its shell (Mantelatto et al. 2007). This deshelling procedure was used for all hermit crabs regardless of shell size. To minimize handling stress, the shell‐less crab was weighed immediately upon removal from its shell, after which the mass of the original shell was recorded and the crab was placed in a small bowl containing seawater and a new shell until the crab entered the shell. Five *P. hirsutiusculus* were provided with a small shell, and five were provided with a large shell. The small shells were of the snail species *Nucella ostrina* with masses ranging from 0.42 to 0.49 g, and the large shells were of the snail species *Nucella lamellosa* with masses ranging from 4.0 to 4.5 g (Figure 3). The small shells used in this experiment were 16.24 ± 0.15 mm (mean ± SE) in length (range: 14.9–17.1 mm), while the large shells were 28.23 ± 0.17 mm in length (range: 26.8–29.6 mm). The volume of the large shells was sufficient for all or nearly all of the body of the crab to retract into the shell, whereas the volume of the small shell was insufficient to allow the front appendages and part of the carapace to retract into the shell. Shells of these two snail species are commonly used by both *P. hirsutiusculus* and *P. granosimanus* in Barkley Sound (Straughan and Gosselin 2014), and shells of these sizes are also frequently used in the field (Straughan and Gosselin 2014; present study). The *N. lamellosa* shells were removed from live hermit crabs in Grappler Inlet, whereas the *N. ostrina* shells were empty when collected from Prasiola Point (48°49′03″ N, 125°09′49″ W). The *N. lamellosa* shells and the *N. ostrina* shells were boiled before being used in the experiment.

Once all 10 hermit crabs had accepted their new shells, each crab was submerged in seawater for 1 h to allow acclimation to the new shells. A hermit crab was considered to have accepted the new shell when it had fully entered the shell and resumed normal movement. Each crab was then gently blot‐dried to remove excess water before being placed in individual containers (11.5 × 11.5 × 12 cm). Containers were placed in two plastic bins (60 × 40.5 × 34.5 cm), six containers per bin; five of the containers each held one crab, and a sixth contained a humidity datalogger (and no crab). The desiccation trials were carried out at 21°C, an intermediate temperature selected based on the preliminary temperature tolerance experiment, which showed that temperatures up to 20°C were non‐lethal for durations of at least 20 h. The VPD level used in the desiccation tolerance experiment was 1.5 kPa, corresponding to a moderately high VPD level recorded during the field monitoring of VPD levels described above. The VPD of ~1.5 kPa was achieved by adjusting the relative humidity in the incubator to 30%–60% with a temperature of 21°C. A small fan was also placed in each bin to ensure air circulation and homogenous temperature and VPD among the containers in the bin. Each bin was then sealed with a lid to obtain a stable relative humidity, and the bins were placed together in the incubator. All crabs were inspected every 2 h until all 10 crabs were inert, at which point the trial was ended and the crabs were submerged in seawater at 11°C for 7 h and then inspected again to determine if each one was dead or alive. The above procedure was carried out four times using different individuals (i.e., four replicate trials), for a total of 20 *P. hirsutiusculus* being tested using a small shell and 20 *P. hirsutiusculus* being tested using a large shell. Thus, each trial consisted of 5 *P. hirsutiusculus* in the small shell treatment and 5 *P. hirsutiusculus* in the large shell treatment (10 individuals per trial; 40 individuals total). For each of the four replicate trials, we compared the percentage of hermit crabs surviving in the small shell and large shell treatments after 4 h of exposure to desiccation. These paired survivorship values (small shell treatment and large shell treatment of each trial) were compared using a paired *t*‐test to determine whether survivorship differed between shell size treatments under identical trial conditions. Hermit crab body mass and shell mass used in the large and small shell treatments, including shell as a percentage of hermit crab body mass, are summarized in Table S2.

### 2.3 Motility

To understand how the use of smaller, lighter shells by *P. hirsutiusculus* influences its motility, the effect of shell size on the motility of this hermit crab was tested. 28 *P. hirsutiusculus* with a claw length of 6–7 mm were collected from Grappler Inlet, and three additional body parameters were also recorded for each individual: shell type, shell length, and crab body mass including the shell. The motility of each crab was examined when being housed in a light shell, and again when housed in a heavy shell. In addition, the motility of each hermit crab was also tested when housed in the shell it was using in the field (original shell), to determine if the shells adopted by hermit crabs in the field allow high levels of motility. This experiment therefore examined the influence of shell mass on motility, with three treatments: two controlled shell mass treatments, light (small) shells and heavy (large) shells, and the original shell used by the crab in the field (not controlled, shell mass determined during the experiment). The average mass of the 28 *P. hirsutiusculus* used in the motility experiment was 0.68 ± 0.02 g (range: 0.45–0.93 g). The large and small shells used in this experiment were the same as those described for the desiccation tolerance experiment. Mass of shells used in the small, large, and original shell treatments and shell mass as a percentage of hermit crab body mass are reported in Table S3. The assigned sequence of shells tested in the first, second, and third motility trial of an individual hermit crab was changed from one crab to another to account for the possible effects of shell type on subsequent motility. Each crab was deshelled using the method described above, beginning with the hand‐warming method and applying the warm water‐soaked cloth method if necessary. To allow the crabs to acclimate to their new shell, each crab was placed in seawater for a minimum of 1 h after accepting its new shell. Before starting a motility trial with a hermit crab, the top of its shell was dried off and a small spot of white ink (Wite‐Out) was applied; this white spot on the shell was readily visible in the recorded videos, making it easier to track the movements of the crab.

The motility trials were carried out in a tray (68 cm × 44 cm) supplied with a continuous flow of seawater and marked with a 4 cm × 4 cm grid. The tray bottom was plexiglass, ensuring a uniform surface across all trials. The tray was surrounded on all sides by a black opaque curtain to provide a consistent light environment for all hermit crabs tested and also to hide all potentially distracting movements of observers. All movements of the hermit crabs were recorded using a video camera installed 95 cm above the tray. Each hermit crab was recorded for 15 min, after which the crab was removed from the tray, gently deshelled, provided with the next shell, and set aside in seawater for at least an hour before its next trial. This was repeated until all crabs tested that day had been tested in their original shell, a heavy shell, and a light shell. The video recordings were subsequently analyzed using the Tracker Online software (Brown et al. 2022; Open Source Physics, version 6.1.0) to quantify path length (crawling distance) traveled and maximum velocity. This video analysis software has previously been found to be effective for tracking and quantifying the recorded movement of objects (Eadkhong et al. 2012; Sirisathitkul et al. 2013). The masses of the three shell types were compared using a Kruskal‐Wallis test, a non‐parametric alternative to one‐way ANOVA. Then, to determine if shell mass affects hermit crab motility, we compared the crawling distance traveled by each hermit crab and their maximum velocity during the 15 min trial between shell mass treatments using a Random Complete Block ANOVA, with individual hermit crabs as the blocking variable, followed by a Tukey HSD multiple comparisons test when an ANOVA was significant.

### 2.4 Divergence in Exoskeleton Mass

*Pagurus hirsutiusculus* and *P. granosimanus* from three field sites were examined to determine if the two hermit crab species differ in exoskeleton mass. In hermit crabs, exoskeleton mass reflects both exoskeleton thickness and the size of the exoskeletal plates; therefore, measuring exoskeleton mass allowed the assessment of the combined influence of these two exoskeletal features. 25 crabs of each species were collected from each of the three field sites. Only crabs with a claw length > 3 mm were used, since the carapace of smaller crabs could not be dissected accurately. The crabs used for exoskeleton mass measurements were the same individuals collected for the analysis of shell size use in the field, described above in Section 2.1. Once returned to the laboratory, crabs were placed in a freezer for a minimum of 5 h to euthanize the crabs. The crabs were then thawed, and claw length, shell length, and shell type were recorded. The crab without the shell was then weighed, as well as the mass of the shell. Finally, the mass of the exoskeleton was assessed by determining the dry mass of two dissected parts of the exoskeleton: the carapace and the right claw. The entire carapace, including the dorsal shield, was carefully dissected and all soft tissue was gently scraped off. The right claw of each crab was then also gently detached at the carpo‐propodal joint, and any soft tissue still attached to the claw was removed with a scalpel. Soft tissue inside the detached claw was not removed. The carapace and claw were then placed in a desiccating oven at 60°C for 3 h, after which each dried carapace and claw was weighed to the nearest 0.0001 g using an analytical balance.

To determine if exoskeleton mass differs between species, the same approach was used as described in Section 2.1, in this case using *t*‐tests to compare the ratio of dry mass of the carapace (CaDM) to body mass (BM) (i.e., CaDM × BM^−1^) between hermit crab species at each of the three field sites, the ratio serving to standardize exoskeleton mass as a function of body mass. In addition, the ratio of dry mass of the claw (ClDM) to BM (i.e., ClDM × BM^−1^) was also compared between species at each site using *t*‐tests. These ratios were compared between species separately for each field site because, although hermit crabs from the three sites likely belong to a broad single population due to their dispersing planktonic larval stage, site‐specific comparisons provide insight regarding the relative contributions of evolutionary morphological adaptation and phenotypic plasticity.

## 3 Results

### 3.1 Shell Size Use in the Field

**FIGURE 4:**
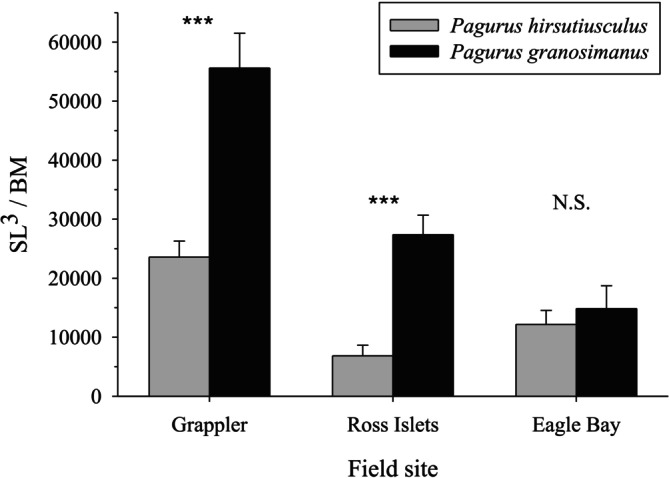
Ratio of shell length^3^ × body mass^−1^ (SL^3^/BM) in *P. hirsutiusculus* and *P. granosimanus* at each of the three field sites. Shell length was cubed to serve as a proxy for shell volume. For each field site, *n* = 25 for each species. Error bars represent SE.

To examine the relationship between shell size used in the field and hermit crab body mass, we compared shell length^3^ relative to body mass between *Pagurus hirsutiusculus* and *Pagurus granosimanus* at each of the three field sites. At two of the three field sites, *P. hirsutiusculus* were using substantially smaller shells, relative to their body mass, than *P. granosimanus* (Figure 4). The ratio of SL^3^ × BM^−1^ was significantly smaller in *P. hirsutiusculus* than in *P. granosimanus* at the Grappler Inlet site (*t*‐test: *t* = 4.934, df = 48, *n* = 50, *p* < 0.001) and Ross Islets site (*t*‐test, adjusted for unequal variances: *t* = 5.475, df = 37, *n* = 50, *p* < 0.001). Shells used by *P. granosimanus* had a SL^3^ × BM^−1^ ratio that was 136% larger (Grappler) and 300% larger (Ross) than the shells used by *P. hirsutiusculus* . At the Eagle Bay site, the SL^3^ × BM^−1^ ratio did not differ significantly between the two hermit crab species (*t*‐test: *t* = 0.591, df = 48, *n* = 50, *p* = 0.557). Hermit crab body mass, shell type, and shell length are reported in Table S4.

### 3.2 Vulnerability to Desiccation

**FIGURE 5:**
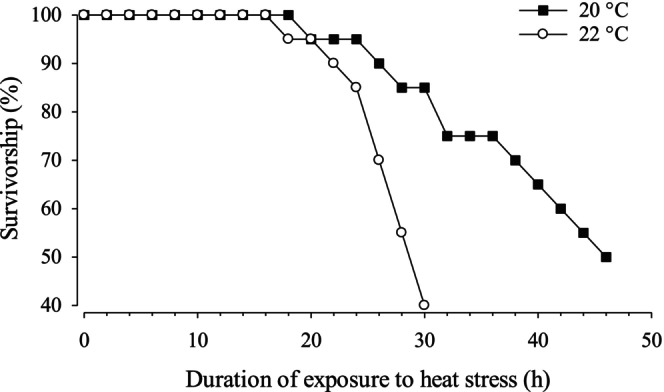
Survivorship of *P. hirsutiusculus* when emersed at ≥ 95% relative humidity at 20°C and 22°C; *n* = 20 in each treatment.

The preliminary experiment, designed to identify the temperature to be used in the desiccation tolerance experiment, revealed a decline in survivorship after 20 h when maintained at 22°C, whereas survivorship remained high for almost 30 h at 20°C (Figure 5). Based on these results, the desiccation tolerance experiment was carried out at the intermediate temperature of 21°C.

**FIGURE 6:**
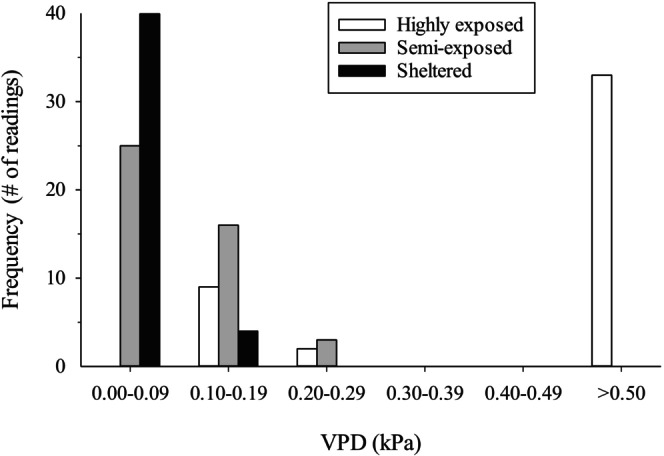
Frequency distribution of VPD readings, recorded at 5 min intervals over a period of 3 h 35 min during low tide on 31 July 2022 in three distinct intertidal microhabitats in Grappler Inlet; *n* = 44 readings in each microhabitat.

VPD monitoring of three intertidal microhabitats available to hermit crabs during low tide at the Grappler Inlet site revealed substantial differences among microhabitats in levels of desiccation; VPD values increased (indicating increased desiccation) with the degree of exposure of the microhabitat (Figure 6 and Table 1).

**TABLE 1:** Maximum VPD recorded during low tide on 31 July 2022 over a period of 3 h 35 min. VPD values are based on relative humidity and temperature measurements recorded by data‐loggers placed in three intertidal microhabitats at Grappler Inlet where hermit crabs are found.

Microhabitat	Amount of exposure	Maximum VPD
Beside flat rock	Very exposed	1.78 kPa
Tucked under large rock	Semi‐exposed	0.25 kPa
Crevasse in rock face	Sheltered (minimally exposed)	0.11 kPa

**FIGURE 7:**
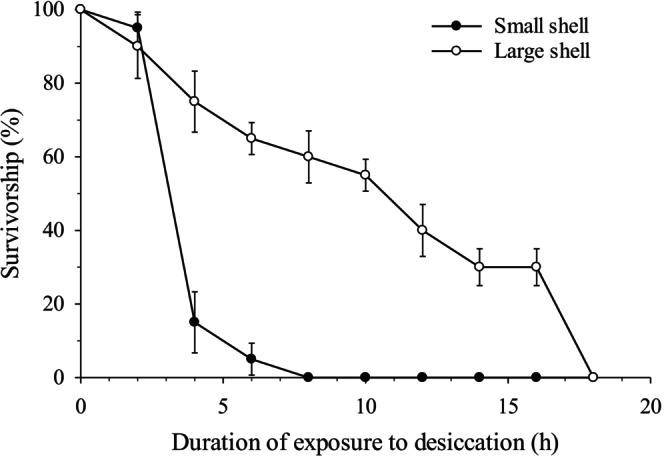
Survivorship of *P. hirsutiusculus* when emersed for 18 h at a VPD of 1.5 kPa. Error bars represent SE; *n* = 4 groups of 5 hermit crabs for each shell size.

The above results of the preliminary thermal tolerance experiment and the field monitoring of VPD levels served as the basis for selecting the conditions to be used in the desiccation tolerance experiment: 21°C and a VPD level of 1.5 kPa. To assess survivorship of *P. hirsutiusculus* during the desiccation tolerance experiment, the probing technique described above was used to assess mortality, as all individuals observed as inert for ≥ 2 h were then found to be dead following the submersion period. Survivorship of *P. hirsutiusculus* individuals using small shells and those using large shells began to diverge after only 4 h (Figure 7), at which time survivorship was significantly lower in *P. hirsutiusculus* using small shells than in individuals using large shells (paired *t*‐test; *t* = 7.35, df = 3, *n* = 4 pairs, *p* = 0.005). After only 4 h of exposure to the desiccating conditions, survivorship was already 400% greater among individuals using large shells than among individuals using small shells.

### 3.3 Motility

**FIGURE 8:**
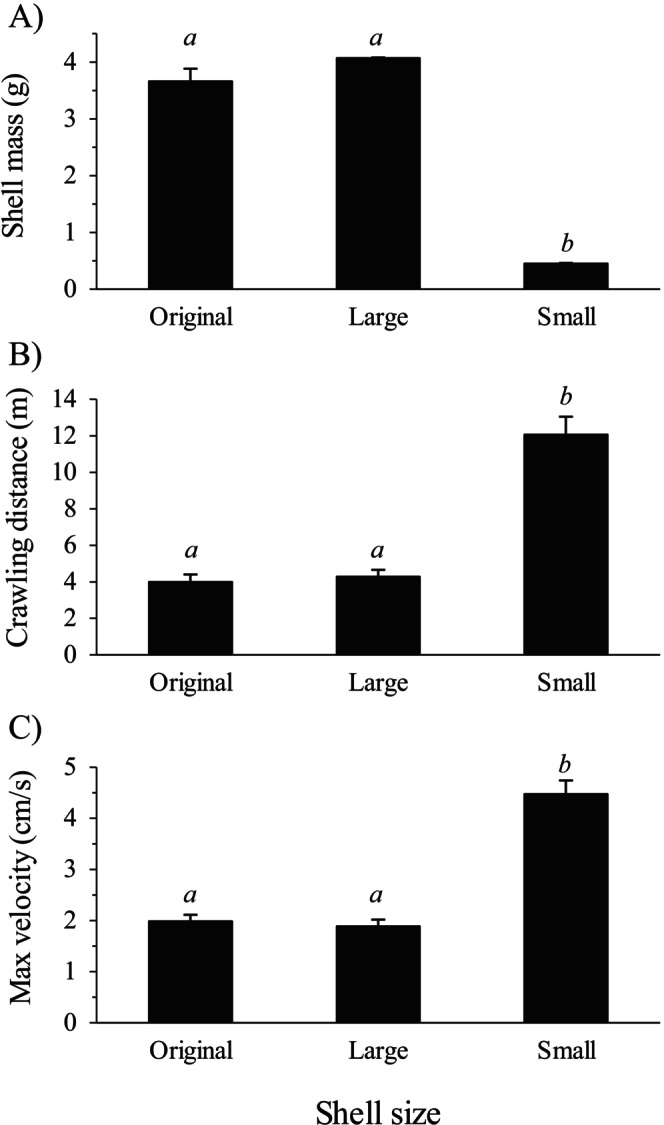
Shell mass of each shell size category used in the motility experiment (A), crawling distance achieved in 15 min by *P. hirsutiusculus* while using a small shell, a large shell, or their original shell (B), and maximum velocity achieved during a 15 min trial by *P. hirsutiusculus* using a small shell, a large shell, or their original shell (C). Maximum velocity was calculated for each individual by taking the average of the five highest velocity values recorded. Error bars represent SE. For (A), (B), and (C) *n* = 28 for each shell treatment. Bars with the same letter are not significantly different, as determined by a Tukey multiple‐comparisons test.

Shell mass differed significantly among the three shell treatments used in the motility experiment (Kruskal–Wallis test: *H* = 57.461, df = 2, *n* = 84, *p* < 0.001), with the mass of the light shells being significantly lower than that of the heavy and original shells, as determined by a pairwise multiple comparison test (Figure 8A). The latter two shell treatments, however, were not significantly different, indicating that the shells used by crabs of this size in the field at the time of this study were similar in mass to the heavy experimental shells. The motility experiment revealed a significant effect of shell mass on distance traveled (Randomized Complete Block ANOVA, using individual hermit crabs as blocks: *F*_2,54_ = 69.134, *n* = 28, *p* < 0.001). Tukey multiple comparisons tests revealed that *P. hirsutiusculus* using a light shell traveled a significantly greater distance than when the same individual was using a heavy shell or its original shell (Figure 8B), but there was no significant difference in crawling distance between the heavy shell and the original shell treatments. When using a light shell, hermit crabs traveled almost three times as far (281%) during the 15 min trials as they did when using a heavy shell. Shell mass also significantly affected the maximum velocity achieved by *P. hirsutiusculus* (RCB ANOVA, using individual hermit crabs as blocks: *F*_2,54_ = 82.847, *n* = 28, *p* < 0.001). Hermit crabs using a light shell achieved a higher velocity than when using a heavy shell or their original shell, but there was no significant difference in maximum velocity between the heavy shell and original shell treatments (Figure 8C). When using a light shell, hermit crabs reached maximum crawling speeds that were more than twice as fast (233% faster) as when they were using a heavy shell.

### 3.4 Divergence in Exoskeleton Mass

**FIGURE 9:**
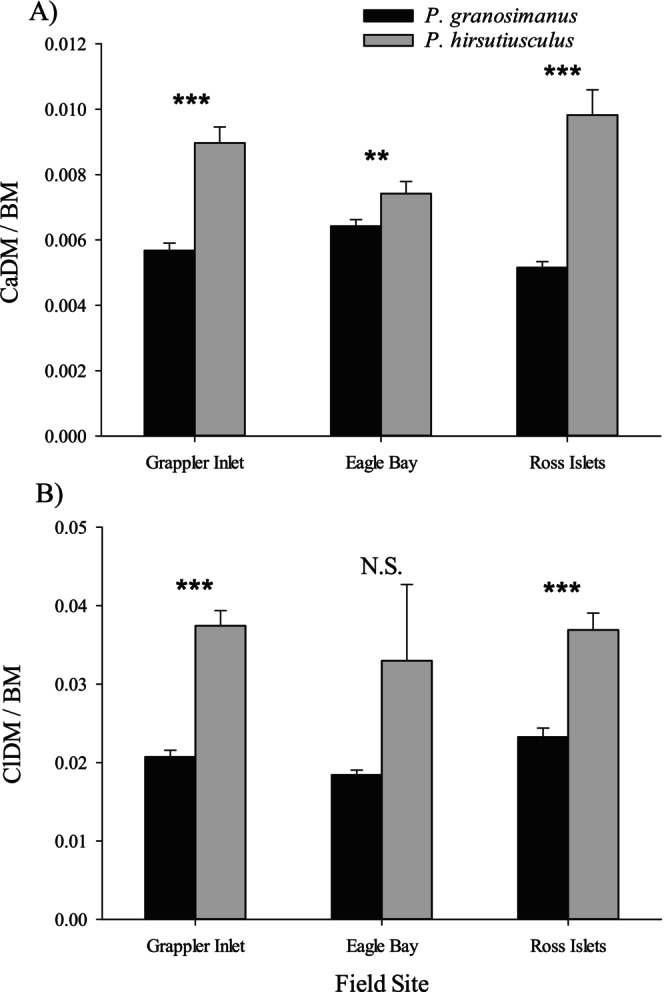
Ratio of dry mass of the carapace × claw length^−3^ (CaDM/CL^3^) (A) and ratio of dry mass of the claw × claw length^−3^ (ClDM/CL^3^) (B) of *P. hirsutiusculus* and *P. granosimanus* collected from each field site. Error bars represent SE. For both (A) and (B): *n* = 25 for each species at each site.

To test for divergence in exoskeleton mass between species, we compared both the dry mass of the carapace and the dry mass of the claw relative to body mass. Information on the body masses of hermit crabs used in this analysis is provided in Table S4. The ratio of dry mass of the carapace to body mass (CaDM × BM^−1^) was significantly higher in *P. hirsutiusculus* than in *P. granosimanus* at all three field sites (Figure 9A): the ratio was 58% higher in *P. hirsutiusculus* at Grappler Inlet (*t*‐test, adjusted for unequal variances: *t* = −6.11, df = 33, *n* = 50, *p* < 0.001), 15% higher at Eagle Bay (*t*‐test, adjusted for unequal variances: *t* = −2.37, df = 36, *n* = 50, *p* = 0.023), and 90% higher at Ross Islets (*t*‐test, adjusted for unequal variances: *t* = −5.89, df = 26, *n* = 50, *p* < 0.001).

The ratio of dry mass of the claw to body mass (ClDM × BM^−1^) was significantly higher in *P. hirsutiusculus* than in *P. granosimanus* at two of the three field sites (Figure 9B): the ratio was 81% higher in *P. hirsutiusculus* at Grappler Inlet (*t*‐test, adjusted for unequal variances: *t* = −7.79, df = 33, *n* = 50, *p* < 0.001), and 59% higher at Ross Islets (*t*‐test, adjusted for unequal variances: *t* = −5.66, df = 36, *n* = 50, *p* < 0.001). The ClDM × BM^−1^ ratio was not quite significantly different between species at Eagle Bay (*t*‐test, adjusted for unequal variances: *t* = −1.49, df = 24, *n* = 50, *p* = 0.149).

## 4 Discussion

### 4.1 Shell Size Use in the Field

At two of the three field sites, *Pagurus hirsutiusculus* used substantially smaller shells, for a given body mass, than *Pagurus granosimanus* , confirming the findings of a previous study (Straughan and Gosselin 2014). The present study revealed that *P. granosimanus* used shells that were 136%–300% larger in volume than the shells used by *P. hirsutiusculus* at the Grappler Inlet and Ross Islets sites. It is interesting, however, that the two species did not use different shell sizes at the Eagle Bay site. This outcome could have been due to constraints in shell availability at the Eagle Bay site, as shell availability can strongly affect shell use by hermit crab populations (Vance 1972; Spight 1977; Bertness 1981a; Halpern 2004). This is supported by the relatively low SL^3^ × BM^−1^ ratio of *P. granosimanus* at Eagle Bay, whereas *P. hirsutiusculus* at Eagle Bay was using shell sizes comparable to conspecifics at the other two field sites. This suggests large shell sizes may not have been available to *P. granosimanus* at the Eagle Bay site.

### 4.2 Functional Consequences of Shell Size

Shell size was found to be associated with functional consequences for *P. hirsutiusculus* . Shell size significantly affected hermit crab vulnerability to desiccation: *P. hirsutiusculus* using shells large enough to shelter the entire body could withstand desiccation for substantially longer periods of time than *P. hirsutiusculus* using smaller shells that could not accommodate the entire carapace or the appendages. When exposed to a VPD of ~1.5 kPa (moderate level of desiccation), survivorship among individuals using small shells quickly dropped below 20%, revealing a high vulnerability to desiccation within 2–4 h of exposure, whereas survivorship remained high for up to 8 h in the same conditions among those using a large shell. Exposure periods of 2–4 h occur often during low tides in Barkley Sound, even for hermit crabs located in the low intertidal zone, making this duration of exposure to desiccating conditions representative of field conditions. The greater survivorship in large shells was undoubtedly due to the larger shells allowing the hermit crab to withdraw more of its body, leaving less of its body directly exposed to air and thus to desiccation. This is consistent with a previous observation that *P. granosimanus* and *Pagurus samuelis* collected from the field, and using shells that do not allow the body to fully withdraw, were more vulnerable to desiccation (Taylor 1981). In addition, large shells may hold more internal water than small shells, further aiding in resisting desiccation (Bertness 1981b).

The VPD level used in the desiccation tolerance experiment (1.5 kPa) is a level that occurs in Barkley Sound in summertime, as recorded during field monitoring at the Grappler Inlet site in the present study, and as previously reported for other intertidal sites in Barkley Sound (Jenewein and Gosselin 2013). In fact, intertidal VPD levels can reach values much higher than 1.5 kPa in Barkley Sound (Jenewein and Gosselin 2013), such that desiccation during summertime low tides would often be a considerable threat to these hermit crab species. As a result, the shell size used by a hermit crab would have a considerable effect on the vulnerability of the crab to ambient aerial conditions during summertime low tides.

The experiment examining the motility of *P. hirsutiusculus* revealed that shell size also has a strong influence on hermit crab motility. A hermit crab inhabiting a light (small) shell could move 2.3 times as fast and 2.8 times as far over a 15 min period than when inhabiting a heavy (large) shell. *Pagurus hirsutiusculus* therefore does gain an advantage of increased motility by selecting smaller shells, as we had initially hypothesized. A similar pattern has been observed in the field for another hermit crab species, *Calcinus californiensis*, where individuals using lighter shells traveled longer distances than those using heavier shells (Alcaraz and García‐Cabello 2017). By facilitating motility, the use of smaller shells by *P. hirsutiusculus* could enhance access to available resources (Tricarico and Gherardi 2006), as well as enhance competitive ability and capacity to avoid predators (Mima et al. 2003; Rosen et al. 2009; Alcaraz and Arce 2017).

The difference in motility between the light and heavy shell treatments was likely due to the lower mass of the light shells, which were substantially lighter to carry and also less bulky than heavy shells. In addition, if hermit crabs were uncomfortable in the smaller shells, this could have motivated them to move rapidly to seek a larger shell (Tricarico and Gherardi 2007; Gherardi and Atema 2005), further increasing crawling speed and distance traveled.

### 4.3 Structural Response: Exoskeleton Mass

An important finding of this study is that exoskeleton mass differs between *P. hirsutiusculus* and *P. granosimanus* , supporting the initial hypothesis that *P. hirsutiusculus* produces a heavier carapace than *P. granosimanus* . The carapace, standardized for body size, was 15%–90% heavier in *P. hirsutiusculus* than in *P. granosimanus* at the three field sites. The analysis of claw mass further confirmed the findings for carapace mass, with standardized claw mass being 59%–81% heavier in *P. hirsutiusculus* than in *P. granosimanus* at two of the three field sites.

Thus *P. hirsutiusculus* , carrying smaller shells that provide less protection, compensate for the use of smaller shells by producing a heavier exoskeleton, as predicted. In *P. granosimanus*, the use of larger shells likely reduces motility and increases energetic cost (Herreid and Full 1986), but provides enhanced protection, allowing for the production of a lighter carapace and claws. *Pagurus hirsutiusculus* , on the other hand, uses shells that are smaller and lighter and thus impose lesser energetic costs (Herreid and Full 1986) and allow greater motility (present study), but leave the animal more vulnerable to stressors (Angel 2000; present study); therefore, *P. hirsutiusculus* invests in a heavier carapace and claws, compensating for the reduced shell protection.

### 4.4 Ecological Implications

The differences in shell size usage and exoskeleton mass suggest ecological implications for these species, particularly with regard to microhabitat use (Bach and Hazlett 2009). Given the increased vulnerability due to the use of small shells, it is expected that *P. hirsutiusculus* would need to relocate away from exposed microhabitats very shortly after low tide emersion of the habitat to more sheltered microhabitats with low VPD levels, such as rock crevices, the underside of microalgae, rocks or shells, and tide pools (Jenewein and Gosselin 2013; present study). Enhanced motility would facilitate this relocation, while the heavier exoskeleton may reduce the vulnerability to predation associated with the use of smaller shells. Additionally, *P. hirsutiusculus* may also mitigate exposure to desiccation stress through clustering with other hermit crabs. Conversely, it would be less urgent for *P. granosimanus* to avoid desiccation stress during the first several hours of a low tide or predators at high tide, and thus this species may be able to move, albeit slowly, to access resources located in exposed microhabitats that could not be explored by hermit crabs using smaller shells. Further studies are needed to examine whether microhabitat use in the field differs between the two species in a way that is consistent with their shell size preferences, and whether *P. hirsutiusculus* is more limited than *P. granosimanus* in the types of microhabitats used, particularly during summertime low tides when desiccation levels are high.

Finally, it is not clear whether the divergent traits of these two species, with differing costs and benefits, are equivalent performance strategies under the same set of selective pressures, or whether the divergence has resulted from *P. granosimanus* experiencing greater threats, and thus requiring greater protection than *P. hirsutiusculus* .

## Author Contributions

**Chloe B. MacLean:** conceptualization (supporting), data curation (lead), formal analysis (equal), investigation (lead), methodology (equal), project administration (supporting), validation (equal), visualization (equal), writing – original draft (lead), writing – review and editing (equal). **Louis A. Gosselin:** conceptualization (lead), data curation (supporting), formal analysis (equal), funding acquisition (lead), methodology (equal), project administration (lead), resources (lead), supervision (lead), validation (equal), visualization (equal), writing – original draft (supporting), writing – review and editing (equal).

## Funding

This work was supported by Thompson Rivers University, UREAP and Natural Sciences and Engineering Research Council of Canada, RGPIN‐2020‐4935.

## Conflicts of Interest

The authors declare no conflicts of interest.

## Supporting information


**Data S1:** ece373044‐sup‐0001‐Supinfo01.docx.

## Data Availability

Data available from the Dryad data repository (https://doi.org/10.5061/dryad.crjdfn3h8).
