# Supplementary material for: Performance Implications of Divergent Shell Size Preferences and Exoskeleton Mass of Two Closely Related Hermit Crabs
Source: Ecol Evol. 2026 Feb 4;16(2):e73044. doi: 10.1002/ece3.73044 (PMC12873454; doi:10.1002/ece3.73044)
Supplement: Supplementary file 1 — Data S1: ece373044‐sup‐0001‐Supinfo01.docx. [file ECE3-16-e73044-s001.docx]

**SUPPLEMENTARY INFORMATION**

**Table S1. Claw length ranges of *Pagurus hirsutiusculus* and *Pagurus granosimanus* from three sites that were used in analyses of shell size usage in the field and exoskeleton mass (n= 25 for each species at each site).**

| **Field Site** | ***P. hirsutiusculus***  **claw length (mm)** | ***P. granosimanus* claw length (mm)** |
| --- | --- | --- |
| Grappler Inlet | 3.55 – 13.75 | 3.30 – 8.85 |
| Ross Islets | 4.50 – 10.20 | 3.15 – 8.60 |
| Eagle Bay | 3.90 – 7.80 | 3.25 – 8.50 |

**Table S2. Average mass of *Pagurus hirsutiusculus* and shells used in the small and large shell treatments of the desiccation tolerance experiment (mean ± SE, range) (n = 20 for each shell treatment).**

| **Shell treatment** | **Hermit crab mass (g)** | **Shell mass (g)** | **Ratio of shell mass to hermit crab body mass %)** |
| --- | --- | --- | --- |
| Small | 0.63 ± 0.03 (0.43–1.00) | 0.45 ± 0.01 (0.42–0.48) | 72.83 ± 3.06 (48.00–109.30) |
| Large | 0.57 ± 0.02 (0.40–0.74) | 4.28 ± 0.04 (4.00–4.50) | 765.01 ± 27.25 (571.43–1000.00) |

**Table S3. Average mass of shells used in the small, large, and original shell treatments of the motility experiment (mean ± SE, range) (n = 28 for each shell treatment). The same *Pagurus hirsutiusculus* were used across treatments, with an average mass of 0.68 ± 0.02 g (0.45–0.93).**

| **Shell treatment** | **Shell mass (g)** | **Ratio of shell mass to hermit crab body mass (%)** |
| --- | --- | --- |
| Small | 0.46 ± 0.01 (0.42–0.49) | 69.18 ± 2.48 (45.16–97.96) |
| Large | 4.07 ± 0.01 (4.04–4.13) | 619.29 ± 23.20 (444.09–897.78) |
| Original | 4.28 ± 0.04 (4.00–4.50) | 765.01 ± 27.25 (571.43–1000.00) |

**Table S4. Average body mass and shell length of *Pagurus hirsutiusculus* and *Pagurus granosimanus* collected from three sites and used in the shell size use in the field and exoskeleton mass analyses. At Grappler Inlet, both species used shells of *Nucella lamellosa* and *Lirabuccinum dirum*. At Ross Islets, both species used shells of *Nucella ostrina*, *L. dirum*, and *Tegula funebralis*; *P. granosimanus* also used *N. lamellosa*, while *P. hirsutiusculus* also used *Littorina sitkana*. At Eagle Bay, both species used shells of *L. dirum* and *T. funebralis*; *P. granosimanus* also used *N. ostrina* (mean ± SE, range) (n = 25 for each species at each site).**

| **Field Site** | **Hermit crab species** | **Body Mass (g)** | **Shell length (mm)** |
| --- | --- | --- | --- |
| Grappler Inlet | *P. hirsutiusculus* | 0.69 ± 0.11 (0.17 – 2.83) | 22.55 ± 0.99 (12.9 – 35.05) |
| Grappler Inlet | *P. granosimanus* | 0.62 ± 0.06 (0.12 – 1.35) | 30.48 ± 0.82 (20.95 – 38.8) |
| Ross Islets | *P. hirsutiusculus* | 0.46 ± 0.07 (0.06 – 1.45) | 12.41 ± 1.11 (6.3 – 24.3) |
| Ross Islets | *P. granosimanus* | 0.68 ± 0.08 (0.10 – 1.40) | 23.73 ± 1.80 (7.10 – 39.00) |
| Eagle Bay | *P. hirsutiusculus* | 0.69 ± 0.06 (0.17 – 1.22) | 18.55 ± 0.97 (11.2 – 29.2) |
| Eagle Bay | *P. granosimanus* | 0.76 ± 0.08 (0.20 – 1.61) | 18.80 ± 1.11 (3.9 – 29.25) |
